# Optimized multiplex PCR-NGS for comprehensive HLA genotyping in Chinese populations: resolving ambiguities at high resolution

**DOI:** 10.3389/fimmu.2025.1551173

**Published:** 2025-06-26

**Authors:** Cuello Garcia Haider, Binbin Sun, Yinfeng Wang, Zhoufan Zhang, Changling Cao, Yiying Zhu, Ouzaouit Abdelhak, Huiqiang Huang, Haitao Liu, Tingya Jiang, Xueping Dong, Yang Zhou, Yu Wu

**Affiliations:** ^1^ School of Life Sciences, Jiangsu University, Zhenjiang, China; ^2^ Nephrology Department, The Affiliated Xuzhou Municipal Hospital of Xuzhou Medical University, Xuzhou, China; ^3^ Biostatistics, Research & Development (R&D), AlloDx Biotech (Shanghai), Co., Ltd, Shanghai, China; ^4^ Pediatrics Department, The Affiliated Xuzhou Municipal Hospital of Xuzhou Medical University, Xuzhou, China

**Keywords:** HLA matching, deep sequencing, next-generation sequencing, multilocus sequence typing, HLA alleles

## Abstract

**Introduction:**

Accurate human leukocyte antigen (HLA) genotyping is critical for organ transplantation to ensure donor-recipient compatibility. Conventional methods, such as sequence-based typing (SBT), often face challenges in resolving allelic ambiguities, particularly in highly polymorphic regions of HLA loci. Therefore, this study aimed to develop 6 locus multiplex primers combined with Next-generation sequencing NGS for high-resolution of long sequenceshigh-resolution sequencing, focusing on improving sequencing depth and reducing costs.

**Methods:**

Multiplex PCR primers targeting HLA-A, -B, -C, -DPB1, -DQB1, -DRB1 loci were designed using high-frequency alleles from public databases. PThe primers were optimized using as reference the sequencing depth across loci. The method was validated using SBT and probe capture‑based targeted next‑generation sequencing to evaluate its approach accuracy. Moreover, 770 samples from Chinese population were further studied to verify the allele frequency adding information about HLA types of this population.

**Results:**

The optimized multiplex PCR-NGS sequencing showed depths within athe target range of 100-1000 with high accuracy determined in the 2ndtwo-digit ,and 4thfour-digit and six-digit HLA typing, with a reliability of ≥ 98%, ≥ 95% and ≥ 95% respectively in both methods.

**Discussion:**

Allele digits in the HLA-class I and II loci. However, in the 6th digit of HLA-C, -DQB1, and -DRB1 the accuracy was 94.74%. The developed multiplex PCR-NGS method offers a reliable, cost-effective approach for high-resolution HLA genotyping, and may be particularly suitable for clinical studies, especially in donor-recipient matching during organ transplantation.

## 1 Introduction

Human leukocyte antigen (HLA) genes exhibit high polymorphism across populations (1), making HLA genotyping critical for clinical research especially in organ transplantation (2, 3). Accurate HLA matching between donors and recipients is crucial to ensure optimal conditions for the recipient (4). Currently, techniques such as Sanger sequencing-based typing (SBT) (5), probe capture-based targeted next-generation sequencing (PCT-NGS) (6), and multiplex PCR-based next generation sequencing (MP-NGS) of HLA loci (7) are used for determining HLA alleles.

In HLA typing, the SBT method was considered the gold standard for HLA genotyping from 1996 until 2016 for the incorporation of next-generation sequencing (NGS) (8, 9); it was widely recognized for accurate matching. SBT has certain limitations, including ambiguity resulting from the combination of two or more different alleles (10); moreover, SBT is designed to amplify short targets of the genome, primarily focusing on exons (11, 12), excluding intron and non-coding regions both important in HLA classification. Additionally, HLA has different polymorphic regions that SBT cannot determine and could be identical in *cis* or *trans* sequencing (13). Thus, accurate HLA typing cannot be achieved in these cases. Prior to the development of more advanced techniques, other methods were designed to identify alleles for HLA loci such as PCR-based HLA typing using sequence-specific primers (SSP) and sequence-specific oligonucleotide probes (SSOP); nonetheless, SSP and SSOP are less detailed compared to SBT (14–16).

In 2011, NGS was considered a novel method for advancing immunogenetics (17); however, it was not until 2016 that researchers demonstrated the functionality and reliability of high-resolution sequencing, heralding the beginning of a new gold standard for HLA typing (8, 18). In recent years, however, PCT-NGS (19) and MP-NGS for HLA genotyping have become the focus of investigations due to their high resolution in HLA typing (20) and their ability to resolve the allelic ambiguity in polymorphic HLA regions. MP-NGS may study large genomic regions of HLA genes including exons, introns, and non-coding regions with high depth, reducing errors in the assignment of alleles conferring high precision and resolving ambiguous calls (21, 22), compared to SBT, SSP, and SSOP, which may ignore important information due to its reliance on short-range PCR. Current multiplex PCR kits designed to study HLA loci are tailored to cover each allele independently. Therefore, their primers cannot be mixed to amplify different loci in the same PCR mixture; in contrast, PCT-NGS has the advantage of probe coverage; moreover, it has been demonstrated to exhibit relatively low standards for DNA quality (19). Nevertheless, PCT-NGS needs a longer experimental process and furthermore requires higher DNA concentrations.

This study is valuable because it describes a stable PCR approach, capable of simultaneously amplifying HLA-A, -B, -C, -DQB1, -DRB1, and -DPB1 loci using a one-tube multiplex PCR setup. Therefore, we developed a high-resolution HLA genotyping assay integrating multiplex PCR and high-fidelity NGS, which markedly enhanced sequencing library efficiency. In addition, this process was initially carried out to optimize the sequencing time and the operation of the PCT-NGS method on the Illumina platform since it is costly and time-consuming. Consequently, MP-NGS has adopted this approach based on the available techniques to develop a better methodology and ensure a reliable amplification of HLA-A, -B, -C, -DQB1, -DRB1, and -DPB1 alleles for individual genotypes in Chinese samples.

## 2 Materials and method

### 2.1 Design of multiplex PCR primers

**Figure 1:**
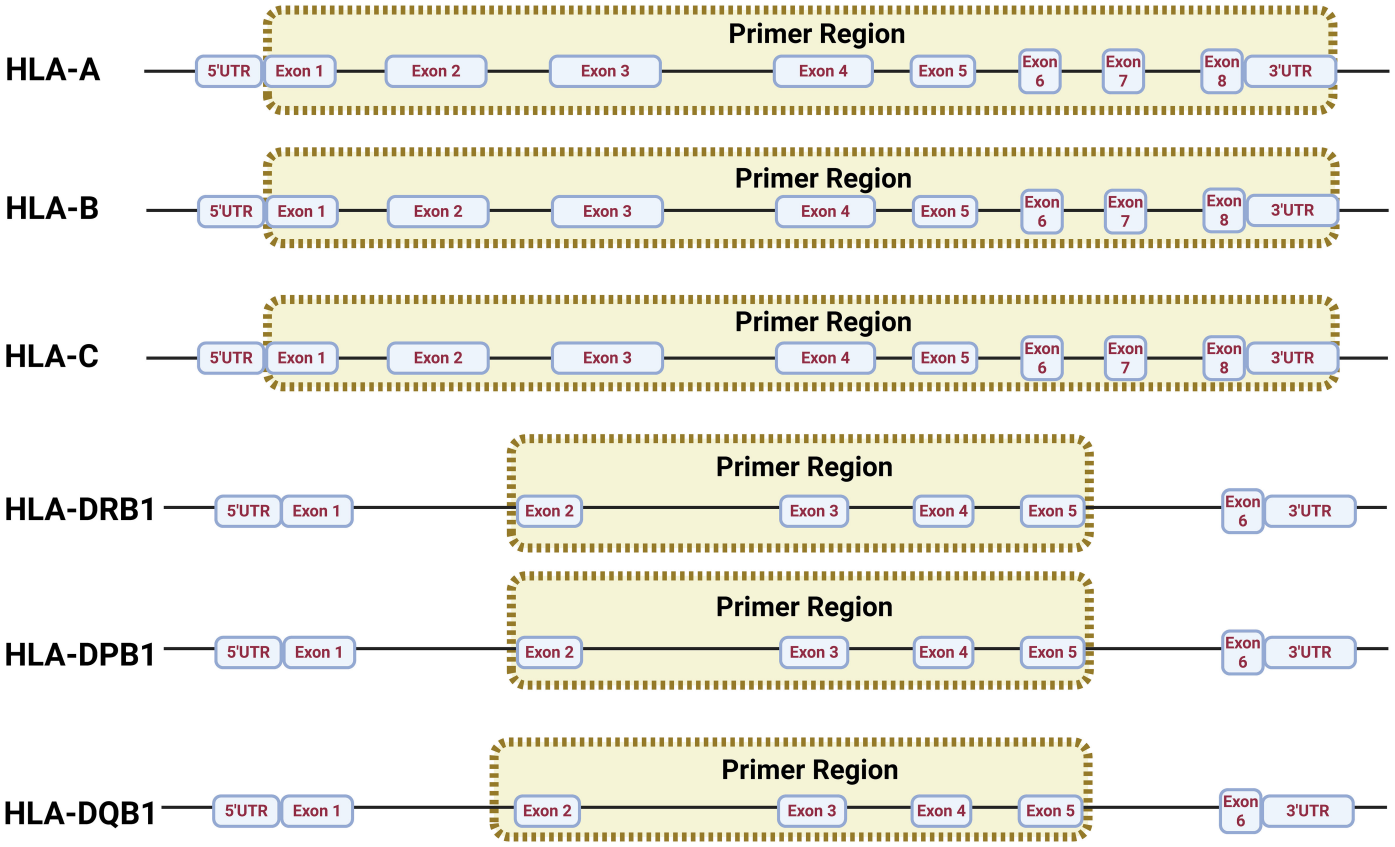
Multiplex PCR primer design principle in HLA loci.

The aim of this experimental study was to design and apply specific primers in a multiplex PCR to amplify the HLA-A, -B, -C, -DRB1, -DQB1, and -DPB1 loci (**Figure 1**), which are important subtypes in the Chinese population (23). Therefore, the primers were designed to amplify samples with high stability, thus avoiding dimers. GenBank, EMBL, and DDBJ, as well as the genome sequence data published on the website (https://www.ebi.ac.uk/ipd/imgt/hla/), were employed to identify high-frequency HLA loci (**Supplementary Table A**). Subsequently, MEGA 11.0 was employed to align the loci and select primer cohorts that can match in high percentage the HLA loci reported for the Chinese population with a frequency of at least 98.5%. In addition, the resulting length designed for the target products was in the range of 2,000–6,000 bp.

### 2.2 Multiplex PCR and sequencing library creation

#### 2.2.1 First step: amplification of HLA loci

**Figure 2:**
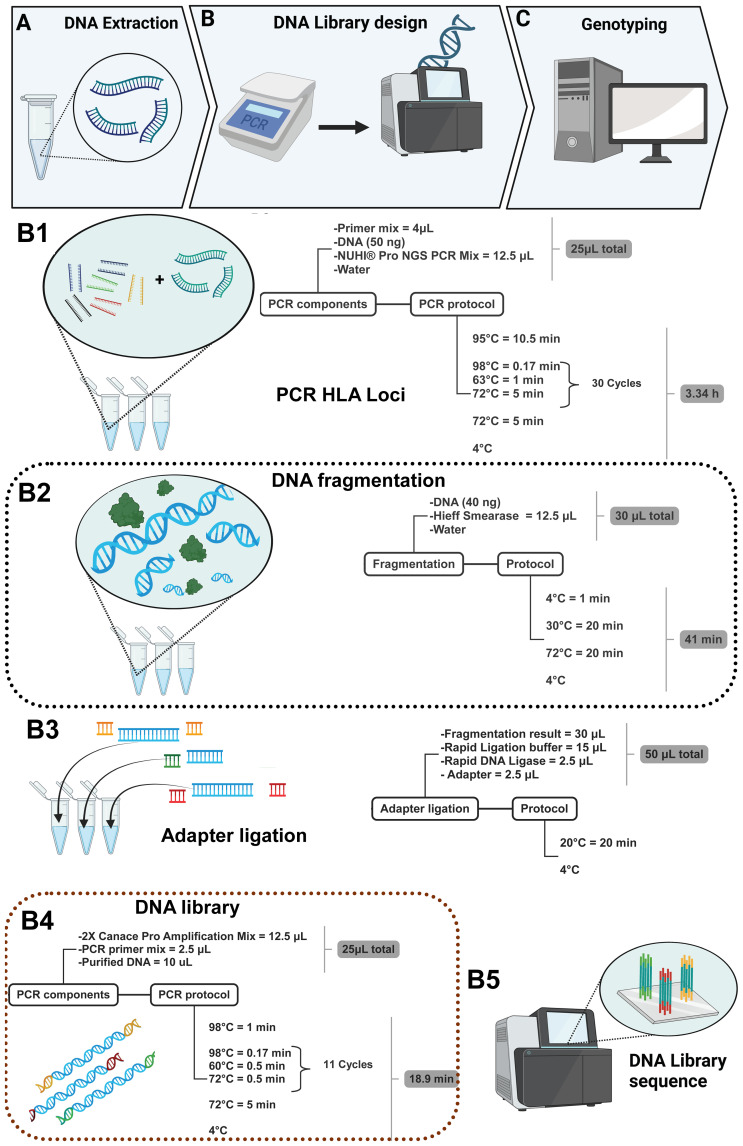
DNA library construction method for six loci in 770 samples.

The multiplex PCR amplification of target amplicons was performed using gDNA, and it was extracted using the QIAamp kit (QIAGEN, Hilden, Germany) according to the manufacturer’s instruction (**Figure 2A**); Qubit 4.0 (Thermo Fisher Scientific, Waltham, Massachusetts, USA) was used to measure the sample DNA concentration. During the preparation of the DNA library (**Figure 2B**), 50 ng of DNA was employed in a 25-μL reaction system and was used to amplify the HLA regions adding 4 μL of the primer mix and 12.5 μL of NUHI^®^ Pro NGS PCR Mix (Xinhai Biotechnology Co., Ltd, Suzhou, China). Primer sets covering HLA type I and HLA type II were mixed into a multiplex PCR primer pool with the following concentrations: HLA-A (0.04 μM), HLA-B (0.1 μM), HLA-C (0.15 μM), HLA-DQB1 (0.18 μM), HLA-DRB1 (0.07 μM), and HLA-DPB1 2-35 (0.04 μM), in accordance with a suitable depth ratio. Multiplex PCR parameters for the first round of target gene amplification were as follows: first step, 95°C for 10.5 min; second step, 98°C for 0.17 min at denaturation, 63°C for 1 min at hybridization, 72°C for 5 min for 30 cycles; third step, 72°C for 5 min at elongation; finally, the sample was maintained at 4°C for storage (**Figure 2B1**).

#### 2.2.2 Second step: DNA fragmentation of HLA loci

After PCR, in a total volume of 30 μL, 40 ng of the PCR product was used in optimal concentration, and 5 μL of Hieff Smearase (YEASEN Biotech Co., Ltd, Shanghai, China) was employed to cleave and fragment the DNA (**Figure 2B2**). Moreover, the PCR conditions for this reaction were 4°C for 1 min, 30°C for 20 min, and 72°C for 20 min.

#### 2.2.3 Third step: adapter ligation in DNA library preparation

The fragmented DNA product with a size of 250–350 bp was obtained, and it was linked with a specific adapter to identify the sample in the mixture during sequencing. Therefore, 15 μL of Rapid Ligation buffer, 2.5 μL of Rapid DNA Ligase, and 2.5 μL of adapter (all from YEASEN Biotech Co., Ltd, Shanghai, China) were added to the product of the second step. Moreover, the adapters were ligated using a temperature of 20°C for 20 min using the thermal cycler (**Figure 2B3**). After the ligation product, the DNA was purified using Hieff NGS^®^ DNA Selection Beads, and thus, another PCR was performed in a total volume of 25 μL; 10 μL of the purified DNA was added to the 12.5 μL of 2× Canace Pro Amplification Mix from Hieff NGS DNA Library Prep Kit 2.0 (YEASEN Biotech Co., Ltd, Shanghai, China) and 2.5 μL of the PCR primer mix from the MGIEasy Universal DNA Library Conversion Kit (App-A) (MGI Tech Co., Ltd, Shenzhen, China) (**Figure 2B4**). After PCR, it was necessary to purify the product again using magnetic beads, and then the concentration of the purified DNA was quantified to confirm the existence of DNA and thus mix the samples to produce the DNA library, considering a range of 6–10 ng/μL as the final concentration to sequence via NGS using the MGI sequencing platform (**Figure 2B5**) and finishing with genotyping (**Figure 2C**).

### 2.3 Evaluation of MP-NGS results

After sample sequencing, the result was filtered of low-quality and contaminated samples; moreover, sequencing junctions were removed using Cutadapt (https://cutadapt.readthedocs.io/en/stable/#cutadapt). These filtered reads were compared to an integrated genome (containing the human genome reference GRCh38, eight MHC haplotypes, and the human genome reference GRCh38) using BWA (http://bio-bwa.sourceforge.net) to order the fragmented amplicons to recover the original sequence amplified. The HLA-LA tool (24) was used for HLA genotyping using the database of HLA-alleles from IPD-IMGT/HLA (https://www.ebi.ac.uk/ipd/imgt/hla/) (24) for allele identification.

### 2.4 Optimization of multiplex PCR conditions and deep homogenization of NGS sequencing

For the purpose of obtaining sufficient information, each primer concentration was quantified automatically using the software HLA-LA by depth count (Equation 1), by genotyping the samples to establish a range of 100–1000× for an accurate assay. Therefore, prior to optimization, the concentration of each primer was 0.2 μM, and this was adjusted until an optimal depth count is achieved; the primer concentration was in the range of 0.04–0.5 μM based on the suggestions of Henegariu et al. (25) when performing a stable multiplex PCR. Therefore, higher depth was corrected by decreasing primer concentration, considering that using an identical primer concentration for amplicons of different lengths may introduce imbalances in the reaction.


(1) $$Depth=\frac{Number\;of\;reads*Average\;reading\;length}{Length\;of\;the\;target\;region}$$

- Number of reads: HLA-LA reads the BAM file and counts how many aligned records are in the target region.
- Average read length: HLA-LA adds up each read in the target region measured in bp and then it is divided by the total number of aligned reads.
- Length of the target region: HLA-LA determines the beginning and end of the region and subtracts: Length = end − start + 1.

### 2.5 Evaluation of SBT

SBT analysis was performed for the purpose of evaluating the reliability of NGS for the genotyped samples. The analysis employs a PCR of HLA class I (A, B, and C) in exons 2 and 3, and of HLA class II (DR, DQ, and DP) in exon 2 (26, 27) of 70 samples. Moreover, Biopython was employed to align the database of IPD-IMGT/HLA with the correspondent sample analyzed. Furthermore, the SNP profile was also determined between the samples and the sequences of the database reference with fewer variations. However, it is necessary to use high-quality sequences to generate this procedure, since Sanger sequencing results with peaks at low levels of fluorescence can produce wrong results (28).

### 2.6 Evaluation of PCT-NGS

Hybridization capture was performed using the ProbeCap^®^ system (Homgen Biotechnology, China), based on DNA probes developed for Illumina and MGI platforms. The same set of 70 samples previously used for the SBT study were applied to this PCT-NGS approach. Therefore, it was necessary for the capture to employ 500 ng of each library, 5 µL of human cot-1 DNA, and 2 µL of MGI blocker; thereafter, this mix was evaporated at 55–60°C; each sample was resuspended in 10 µL of hybridization buffer (HYB-Buffer), 2 µL of enhancer, and 2 µL of probes thereafter; and water was added until a total volume of 16 µL was reached. The hybridization protocol was as follows: 95°C for denaturation for 5 min and then 65°C for 3 h for the respective hybridization.

When the hybridization protocol ended, it was necessary to add 16 µL of hybridization beads; thereafter, each sample was incubated at 65°C for 30 min with mixing every 10 min to capture the biotinylated probes hybridized to the target DNA. subsequently washing protocol was necessary to pre hot the Wl and S-W at 65°C, following 120 µL of WI was added for 10 s, later 150 µL of S-W for 5 min, thereafter 150 µL of WI , WII, WIII were added for 10 s, considering take each wash before adding the next respectively, and then resuspend the beads in 23 µL of ddwater. The product was amplified (POST-PCR) using 25 µL of 2× HIFI enzyme, 2 µL of adapters, and 23 µL of the microbeads suspended in dd water; thereafter, this mix was amplified using the PCR protocol of 98°C for 45 s, 98°C for 15 s, 50°C for 30 s, 72°C for 30 s for 12 cycles, and 72°C for 1 min, considering a 4°C hold. After PCR, it was necessary to perform a new purification using DNA clean beads by adding 25 µL of water to elute the sample from microbeads.

### 2.7 Statistics

R-statistics 4.4.3 was employed for statistical analysis using the depth count of the sequence to compare pre- and post-optimization, with each value obtained by the HLA-LA genotyping of MP-NGS; the Wilcoxon signed-rank test (*p* < 0.05) was used to study the significant differences of each locus of 40 random samples. Furthermore, ggplot2 version 3.5.1 was selected for illustrating a box-and-whisker plot to visualize their distribution. Moreover, the HLA class I and II loci frequencies were determined by counting via the same program using 770 patients requiring organ transplantation; in addition, samples were provided from the databank of AlloDx (Shanghai) Biotech Co., Ltd., and rare alleles were determined using the web site http://www.allelefrequencies.net, which includes HLA frequency data. Furthermore, homozygosity and heterozygosity assessment was rigorously performed for all samples included in the frequency analysis. This was ensured by maintaining sufficient sequencing depth and high-quality thresholds to decrease allelic dropout and sequencing errors (29); in addition, Integrative Genomics Viewer (IGV) was used to validate the results of the invalidation set. Additionally, the accuracy of six-locus NGS genotyping was determined using Equation 2 with SBT and PCT-NGS results, and the reliability of the methodology has been verified using 70 randomly selected samples in parallel with MP-NGS.


(2) $$\;Accuracy=\;\frac{number\;of\;concordant\;allele\;\left(MP-NGS=SBT\;or\;PCT-NGS\right)}{concordant\;allele+discordant\;allele}$$

## 3 Results

### 3.1 Multiplex PCR primers and experimental optimization

**Figure 3:**
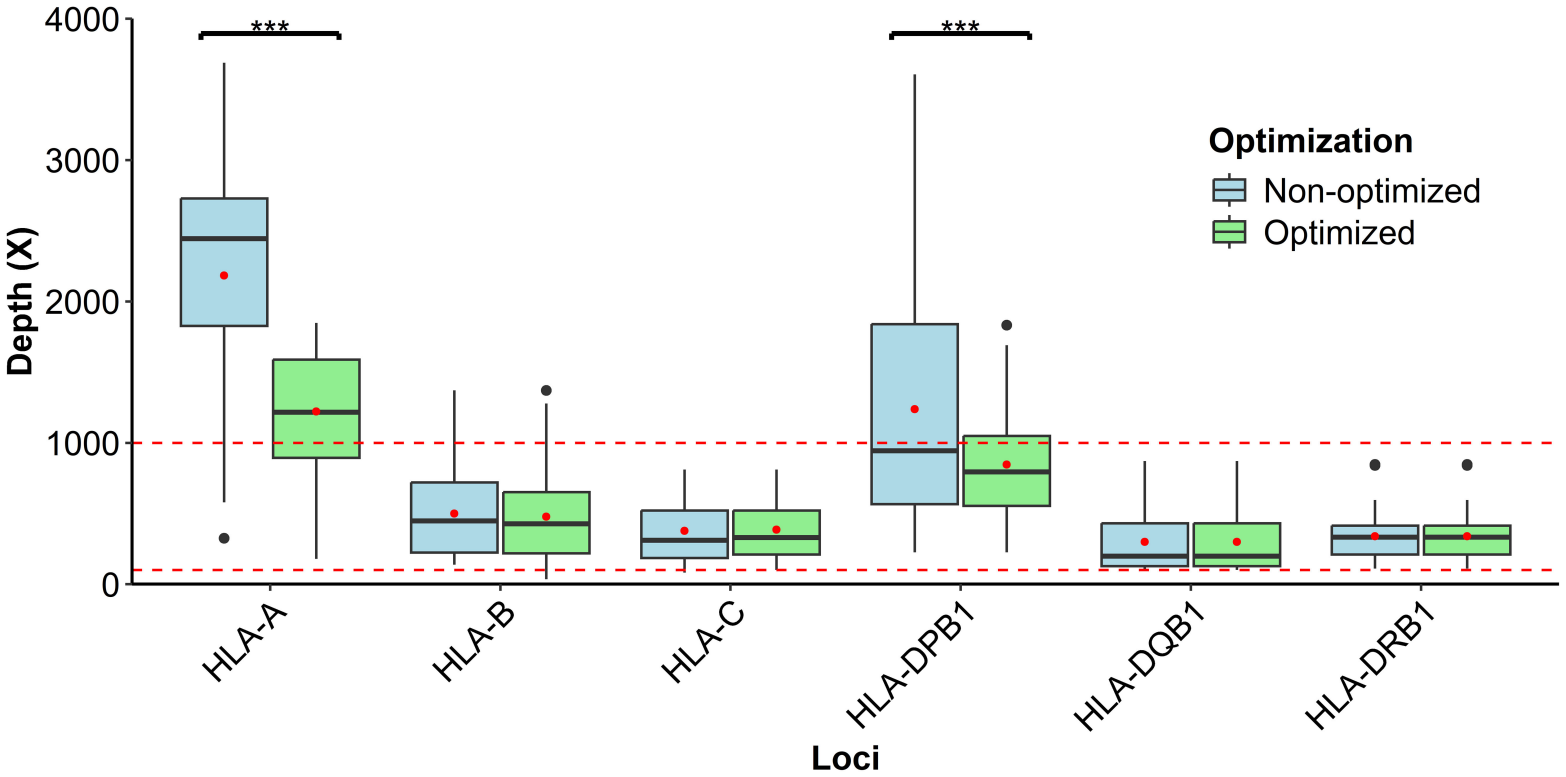
Depth distribution analysis of HLA primer optimization within the defined interval permissible of 100–1,000×, considering that each red point inside the box is the respective average study of loci; moreover, asterisks represent highly significant *p*-values (*p* < 0.01).

Prior to formal sample validation, we performed normalization adjustments to the sequencing depth through the primer concentrations in the multiplex PCR primer pool and the initial DNA input. The analysis of Wilcoxon signed‐rank test demonstrated the influence of altering primer concentration on the measured values for HLA-A (2,444.04 vs. 1,215.59, *p*-value = 1.179e^−05^) and HLA-DPB1 (1,032.43 vs. 794.41, *p*-value = 0.00244). However, no significant differences were observed in the HLA-B (447.56 vs. 427.10, *p*-value = 0.4227), HLA-C (310.35 vs. 330.09, *p*-value = 0.83), HLA-DQB1 (198.12 vs. 196.12, *p*-value = 0.86), and HLA-DRB1 (332.17 vs. 334.171, *p*-value = 0.83) loci. Moreover, our result showed a depth range within 100–1,000 for the multiplex PCR (**Figure 3**). Therefore, post-optimization, a significant decrease in depth was observed in both HLA-A and HLA-DPB1 loci, although HLA-A was out of the established limit. On the other hand, the loci HLA-B, HLA-C, HLA-DQB1, and HLA-DRB1 remained similar before and after modification, and despite variations in primer concentration at loci B, C, DQB1, and DRB1, the sequencing depth remained within the optimal range for robust data analysis.

For the time employed to perform the HLA genotyping results, it was determined that for the library preparation, the time required was 5.67–6 h, while sequencing and bioinformatic analysis were generated at ~28 h. Consequently, the total time used to finish the experiment was 33.67–40 h (**Table 1**), considering that the requisite time depended on the sample size analyzed. Thus, this time lapse may faithfully explain a group of ~5 samples.

**Table 1:** Timing information for post-PCR to sequencing.

Processing time	Step	Time
Library Preparation	Multiplex PCR	3.5 h
	DNA fragmentation	0.75 h
	Native barcode ligation	0.42 h
	DNA purification	1 h
	Total library prep time	5.67 h
Sequencing and Analysis	Sequencing	24 h
	Base calling, demultiplexing, consensus, and genotyping	4 h
	Total sequencing and analysis time	28 h
Total Time		33.67 h

### 3.2 Comparison of MP-NGS results on PCT-NGS and SBT results

**Figure 4:**
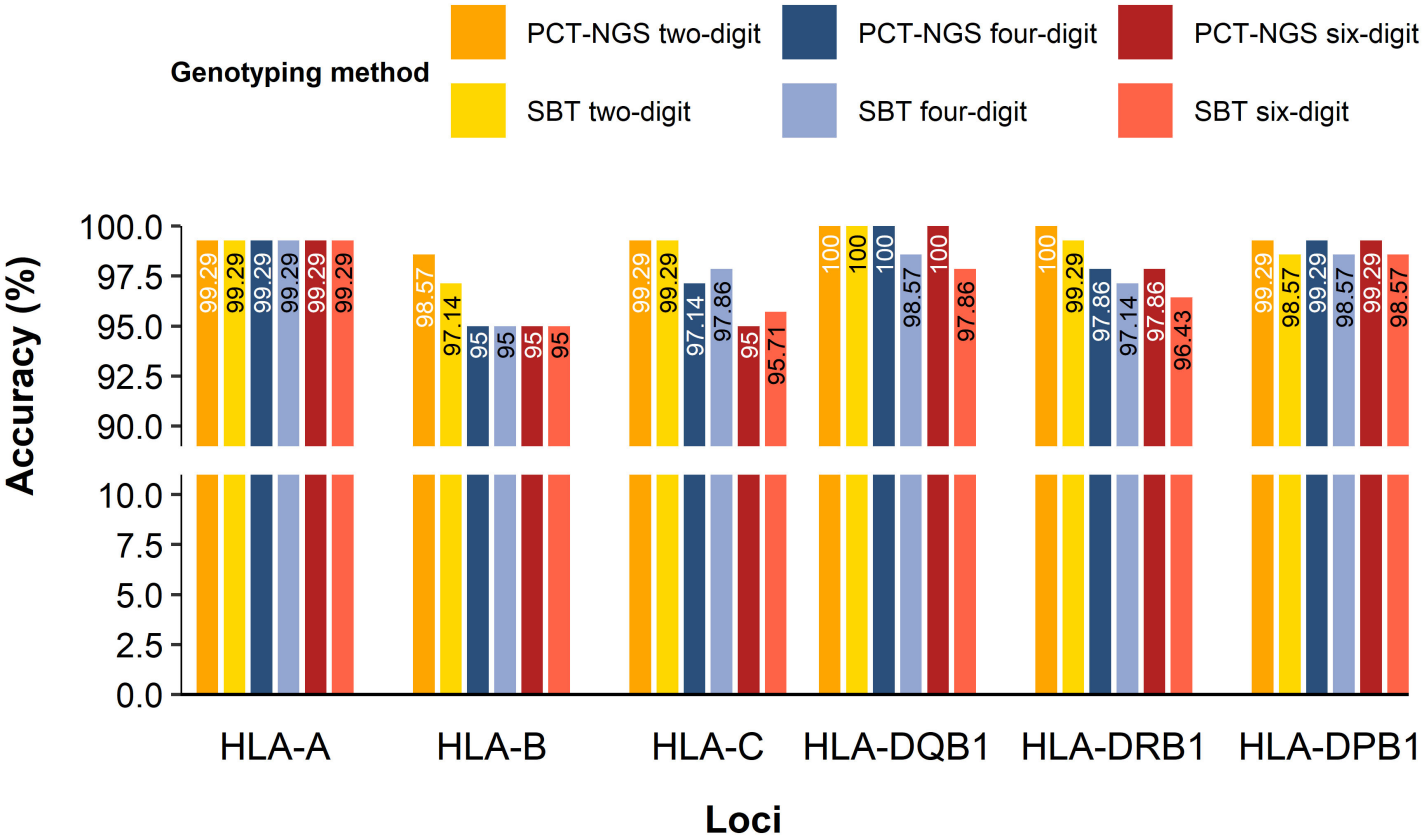
HLA class I and II accuracy for MP-NGS genotyping for two-digit, four-digit, and six-digit.

The comparison of 70 samples mapping using NGS vs. SBT and NGS vs. PCT-NGS determined the accuracy of HLA loci genotyped for MP-NGS methodology. HLA-A showed a concordance rate of 99.29% in both MP-NGS vs PTC-NGS and MP-PCR vs SBT comparisons, across two-digit, four-digit, and six-digit typing resolutions. HLA-B presented different accuracies in different methods; for PCT-NGS, the two-digit typing resolution showed 98.57% confidence. However, SBT reached 97.14%; moreover, at the four-digit HLA typing, both methods showed 95% accuracy, and the six-digit reliability remained at 95% for both methods. For HLA-C, two-digit accuracy was 99.29% for both methods, at the four-digit, PCT-NGS describes 97.14% of confidence, whereas SBT slightly outperformed it with 97.86%; in the six-digit, PCT-NGS showed 95% concordance and SBT showed 95.71%. In class II HLA loci, specifically HLA-DQB1, PCT-NGS showed 100% confidence in the two-digit, four-digit, and six-digit. Using the SBT method, the two-digit had 100% confidence, the four-digit had 98.57% confidence, and the six-digit had 97.86% confidence. For HLA-DRB1, the two-digit showed 100% matching using the PCT-NGS method; however, SBT results described 99.26% accuracy for the same digit; at the four-digit, accuracy was 97.86% using PCT-NGS and 97.14% using SBT, while the six-digit had an accuracy of 97.86% with PCT-NGS and 96.43% with SBT. For HLA-DPB1, 99.29% precision was found in two-digit, four-digit, and six-digit using PCT-NGS; moreover, the reliability of SBT for two-digit, four-digit, and six-digit showed 98.57% (**Figure 4**). These results confirm a high overall reliability of MP-NGS genotyping, with small variations between traditional SBT and PCT-NGS methods across loci and resolution levels.

Consequently, allele frequencies for PCT-NGS, SBT, and MP-NGS alleles were compared (**Table 2**), with few exceptions; NGS provided unambiguous allele assignments at the three-field level at high accuracy within the permitted limits. Ambiguities observed were among the highly polymorphic loci. At HLA-A, a single allele mismatch was observed. For HLA-B, six alleles differed using different methods; however, one allele observed was matched using PCT-NGS, but SBT showed an ambiguous result, with other alleles, analyzed by SBT, showing concordant alleles, but the alleles diverged using PCT-NGS. For HLA-C, six alleles had ambiguous NGS assignments; moreover, sample number 10 differed from SBT. However, its result matched with that of PCT-NGS, and sample number 12 typed with SBT and MP-NGS described a perfect match, but showed mismatch using PCT-NGS.

**Table 2:** Alleles with differences between SBT, PCT-NGS, and MP-NGS analysis.

#	MP-NGS		SBT		PCT-NGS	
	Allele 1	Allele 2	Allele 3	Allele 4	Allele 5	Allele 6
1	A*33:03:01	A*30:13	A*33:03:01	A*68:18N	A*33:03:01	A*31:01:02
2	B*46:01:01	B*14:02:01	B*46:01:01	B*46:01:01	B*46:01:01	B*14:02:01
3	B*13:02:01	B*15:34	B*13:02:01	B*15:400N	B*13:02:01	B*15:01:01
4	B*18:01:01	B*40:83	B*18:01:01	B*07:02:01	B*18:01:01	B*40:06:01
5	B*35:01:01	B*35:01:01	B*35:01:01	B*40:256N	B*35:01:01	B*40:06:01
6	B*40:01:01	B*40:130:02	B*40:01:01	B*40:506N	B*40:01:01	B*40:06:01
7	B*13:01:01	B*13:01:01	B*13:01:01	B*40:506N	B*13:01:01	B*40:06:01
8	B*40:20	B*46:01:01	B*40:20	B*46:01:01	B*40:06:01	B*46:01:01
9	B*07:05:01	B*40:120	B*07:05:01	B*40:506N	B*07:05:01	B*40:06:01
10	C*01:02:01	C*01:02:01	C*01:02:01	C*01:02:02	C*01:02:01	C*01:02:01
11	C*02:02:03	C*03:04:01	C*02:02:02	C*03:04:01	C*02:02:02	C*03:04:01
12	C*03:04:26	C*14:02:01	C*03:04:26	C*14:02:01	C*03:04:01	C*14:02:01
13	C*03:02:01	C*03:151	C*03:02:01	C*03:316N	C*03:02:01	C*03:03:01
14	C*12:02:01	C*14:02:02	C*12:02:01	C*14:02:01	C*12:02:01	C*14:02:01
15	C*04:140	C*06:02:01	C*04:09N	C*06:02:01	C*06:02:01	C*06:02:01
16	C*01:67	C*03:04:01	C*07:02:01	C*03:04:01	C*01:02:01	C*03:04:01
17	C*01:67	C*08:03:01	C*01:67	C*08:03:01	C*01:02:01	C*08:03:01
18	DQB1*06:01:01	DQB1*06:02:01	DQB1*06:02:01	DQB1*06:02:01	DQB1*06:01:01	DQB1*06:02:01
19	DQB1*03:01:01	DQB1*03:03:02	DQB1*03:03:02	DQB1*03:03:02	DQB1*03:01:01	DQB1*03:03:02
20	DQB1*05:01:01	DQB1*05:03:01	DQB1*05:01:45	DQB1*05:03:01	DQB1*05:01:01	DQB1*05:03:01
21	DRB1*08:03:02	DRB1*12:02:01	DRB1*08:03:02	DRB1*12:02:02	DRB1*08:03:02	DRB1*12:02:01
22	DRB1*12:01:01	DRB1*12:02:01	DRB1*12:01:01	DRB1*12:01:01	DRB1*12:01:01	DRB1*12:02:01
23	DRB1*03:01:01	DRB1*04:09	DRB1*03:01:01	DRB1*04:20	DRB1*03:01:01	DRB1*04:05:01
24	DRB1*15:01:01	DRB1*15:02:01	DRB1*15:01:01	DRB1*04:20	DRB1*15:01:01	DRB1*15:01:01
25	DRB1*04:24	DRB1*09:01:02	DRB1*04:20	DRB1*09:01:02	DRB1*04:05:01	DRB1*09:01:02
26	DPB1*02:01:02	DPB1*05:01:01	DPB1*02:01:02	DPB1*02:01:02	DPB1*02:01:02	DPB1*05:01:01
27	DPB1*100:01	DPB1*02:01:02	DPB1*02:01:02	DPB1*02:01:02	DPB1*05:01:01	DPB1*02:01:02

In the HLA-DQB1 locus, three ambiguous alleles were found, and although those ambiguous NGS assignments were confirmed in parallel with SBT, using the PCT-NGS method, the MP-NGS results matched. For HLA-DRB1, which is the most polymorphic of the class II group, there were five ambiguous NGS results; four alleles presented mismatches using SBT as reference; moreover, the allele at position 25 was different using both methods. For HLA-DPB1, two ambiguous mismatches were determined; the allele comparison at position 26 showed a mismatch using SBT; however, using PCT, it was possible to observe a respective match.

Upon analysis of the results, the observed ambiguities were attributed to the causes detailed in **Table 3**. For the HLA-A locus, ambiguities were associated with PCR-induced artifacts. In the case of HLA-B, the ambiguities resulted from inserted sequences detected in the SBT analysis, PCR-induced artifacts due to amplification cycles, allele-specific amplification bias, and erroneous allele calls. For the HLA-C locus, regions not covered by primers, particularly at the UTR boundaries, as well as PCR-induced artifacts and incorrect allele assignments were identified as contributing factors. At the HLA-DQB1 locus, discrepancies in SBT were attributed to SNPs likely introduced by PCR artifacts. For HLA-DRB1, both regions not covered by SBT and PCR-induced artifacts were observed. In the case of HLA-DPB1, ambiguities were also linked to coverage gaps and PCR-induced artifacts.

**Table 3:** Differences analyzed in the ambiguous alleles of the 70 HLA samples.

#	MP-NGS	SBT	PCT-NGS	Affected region	Cause
1	A*30:13	A*68:18N	A*31:01:02	Exon 2	PCR-induced artifacts
2	B*14:02:01	B*46:01:01	B*14:02:01	Exon 2	Sequence insertion
3	B*15:34	B*15:400N	B*15:01:01	Exon 3	PCR-induced artifacts
4	B*40:83	B*07:02:01	B*40:06:01	Exons 2 and 3	PCR-induced artifacts
5	B*35:01:01	B*40:256N	B*40:06:01	Exons 2 and 3	Quantitative imbalance in multiplex PCR amplification
6	B*40:130:02	B*40:506N	B*40:06:01	Exon 3	PCR-induced artifacts
7	B*13:01:01	B*40:506N	B*40:06:01	Exon 2	PCR-induced artifacts
8	B*40:20	B*40:20	B*40:06:01	—————–	Erroneous allele call
9	B*40:120	B*40:506N	B*40:06:01	Exon 3	PCR-induced artifacts
10	C*01:02:01	C*01:02:02	C*01:02:01	Introns	Region not covered
11	C*02:02:03	C*02:02:02	C*02:02:02	Exon 3	PCR-induced artifacts
12	C*03:04:26	C*03:04:26	C*03:04:01	Intron 2	PCR-induced artifacts
13	C*03:151	C*03:316N	C*03:03:01	Exons 2 and 3	PCR-induced artifacts
14	C*14:02:02	C*14:02:01	C*14:02:01	Exon 3	PCR-induced artifacts
15	C*04:140	C*04:09N	C*06:02:01	Exon 2	PCR-induced artifacts
16	C*01:67	C*07:02:01	C*01:02:01	Exon 2	Erroneous allele call and PCR-induced artifacts
17	C*01:67	C*01:67	C*01:02:01	UTR	Region not covered
18	DQB1*06:01:01	DQB1*06:02:01	DQB1*06:01:01	Exon 2	PCR-induced artifacts
19	DQB1*03:01:01	DQB1*03:03:02	DQB1*03:01:01	Exon 2	PCR-induced artifacts
20	DQB1*05:01:01	DQB1*05:01:45	DQB1*05:01:01	Exon 2	PCR-induced artifacts
21	DRB1*12:02:01	DRB1*12:02:02	DRB1*12:02:01	Out Exon 2	Region not covered
22	DRB1*12:02:01	DRB1*12:01:01	DRB1*12:02:01	Out Exon 2	Region not covered
23	DRB1*04:09	DRB1*04:20	DRB1*04:05:01	Exon 2	PCR-induced artifacts
24	DRB1*15:02:01	DRB1*04:20	DRB1*15:01:01	Exon 2	PCR-induced artifacts
25	DRB1*04:24	DRB1*04:20	DRB1*04:05:01	Exon 2	PCR-induced artifacts
26	DPB1*05:01:01	DPB1*02:01:02	DPB1*05:01:01	Exon 2	PCR-induced artifacts
27	DPB1*100:01	DPB1*02:01:02	DPB1*05:01:01	Exon 2	PCR-induced artifacts and region not covered

### 3.3 Frequencies in the Chinese population

#### 3.3.1 Allelic frequency of HLA class I and II

**Figure 5:**
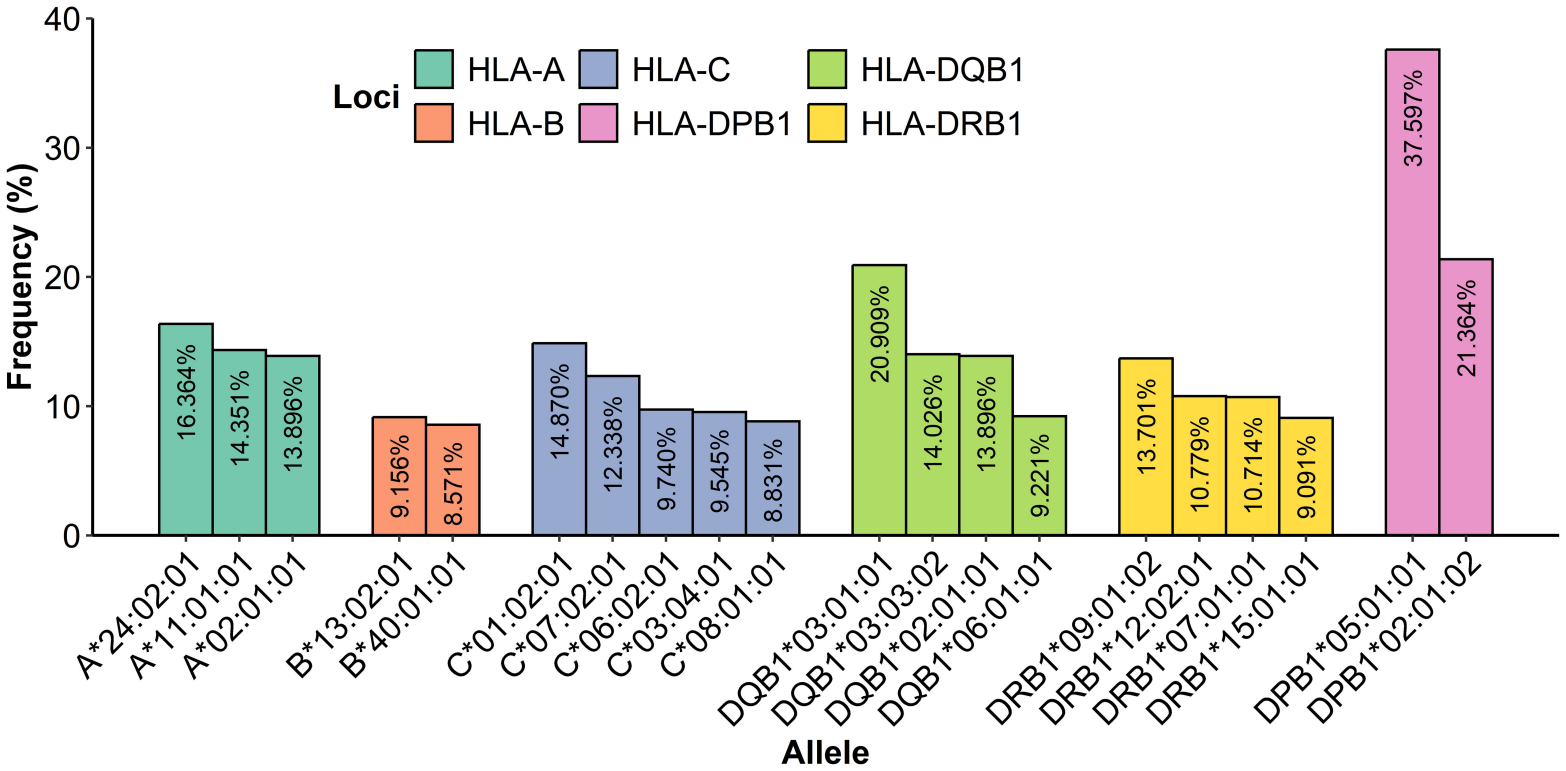
Frequency determination of alleles from HLA class I and class II most presented in 770 samples.

Regarding the frequency analysis of 770 genotyped patients (**Figure 5**), a total of 13 alleles showed high frequency: 5 were identified for HLA class I and 8 for HLA class II. HLA-A presented three alleles with a frequency above 10%: A*24:02:01 at 16.36%, A*11:01:01 at 14.35%, and A*02:01:01 at 13.90%. Additionally, for HLA-C, two alleles were identified with a frequency above 10%: C*01:02:01 at 14.87% and C*07:02:01 at 12.34%; HLA-B was observed as the most polymorphic with 97 different allelic specificities, and the allele B*13:02:01 was the most frequent at 9.29%.

In class II, the locus HLA-DPB1 showed two alleles with a frequency above 10%: DPB1*05:01:01 at 37.60% and DPB1*02:01:02 at 21.36%; notably, the allele DPB1*05:01:01 was the most frequent with 579 repetitions, and HLA-DQB1 showed three alleles with a frequency above 10%: DQB1*03:01:01 at 20.91%, DQB1*03:03:02 at 14.03%, and DQB1*02:01:01 at 13.90%. Additionally, HLA-DRB1 presented three alleles with a frequency above 10%: DRB1*09:01:02 at 13.70%, DRB1*12:02:01 at 10.78%, and DRB1*07:01:01 at 10.71%. Furthermore, there was evidence that 18 patient alleles for A*24:02:01, 14 patient alleles for C*01:02:01, 113 patient alleles for DPB1*05:01:01, 40 patient alleles for DQB1*03:01:01, and 28 patient alleles for DRB1*09:01:02 were homozygous carriers. A complete table with all of the HLA class I and II frequencies is given in the **Supplementary Materials** (**Table B**).

A rare allele study presented alleles A*24:260, C*03:151, C*03:231, C*04:140, C*06:143, and C*12:55, which were the most observed (**Table 4**); moreover, the HLA-C locus presented a more rare polymorphism with 26 different alleles, higher than that of HLA-B with 16 and HLA-A with 9, assuming that locus C includes a larger number of rare alleles.

**Table 4:** Rare allele frequency determined in HLA class I.

#	HLA-A	%	HLA-B	%	HLA-C	%
1	A*24:260	0.1786	B*15:188	0.0893	C*03:151	0.1786
2	A*02:255	0.0893	B*27:05:14	0.0893	C*03:231	0.1786
3	A*02:543	0.0893	B*35:09:02	0.0893	C*04:140	0.1786
4	A*11:147	0.0893	B*35:197	0.0893	C*06:143	0.1786
5	A*11:88	0.0893	B*37:50	0.0893	C*12:55	0.1786
6	A*31:01:07	0.0893	B*46:12	0.0893	C*01:02:32	0.0893
7	A*02:112	0.0893	B*40:219	0.0893	C*01:102	0.0893
8	A*25:30	0.0893	B*51:157	0.0893	C*03:04:03	0.0893
9	A*30:11:02	0.0893	B*54:04	0.0893	C*03:04:38	0.0893
10			B*54:16	0.0893	C*03:116:01	0.0893
11			B*58:16:02	0.0893	C*03:99	0.0893
12			B*58:34	0.0893	C*03:132	0.0893
13			B*40:53	0.0893	C*04:178	0.0893
14			B*46:03	0.0893	C*06:02:43	0.0893
15			B*46:13:03	0.0893	C*06:132:01	0.0893
16			B*54:30	0.0893	C*06:147	0.0893
17					C*08:08:02	0.0893
18					C*08:16:01	0.0893
19					C*08:72:01	0.0893
20					C*12:49	0.0893
21					C*14:02:05	0.0893
22					C*14:09	0.0893
23					C*14:17	0.0893
24					C*14:18	0.0893
25					C*14:58	0.0893
26					C*14:69	0.8929

The rare analysis described that the alleles DRB1*09:05 and DQB1*03:69 were the most frequent at 0.268% and 0.179%, respectively, considering that the DRB1 locus was the most polymorphic locus of HLA class II with 16 rare alleles, and DQB1 presented 10 different rare alleles (**Table 5**).

**Table 5:** Rare allele frequency determined in HLA class II.

#	HLA-DPB1	%	HLA-DQB1	%	HLA-DRB1	%
1	DPB1*05:01:02	0.0893	DQB1*03:69	0.1786	DRB1*09:05	0.2679
2	DPB1*394:01	0.0893	DQB1*02:47	0.0893	DRB1*04:107	0.0893
3			DQB1*02:50	0.0893	DRB1*04:116	0.0893
4			DQB1*02:54	0.0893	DRB1*04:152	0.0893
5			DQB1*03:10:02	0.0893	DRB1*04:77	0.0893
6			DQB1*03:112	0.0893	DRB1*04:86	0.0893
7			DQB1*03:57	0.0893	DRB1*07:25	0.0893
8			DQB1*02:38	0.0893	DRB1*07:50	0.0893
9			DQB1*06:19:01	0.0893	DRB1*03:105	0.0893
10			DQB1*06:67	0.0893	DRB1*11:01:13	0.0893
11					DRB1*11:130	0.0893
12					DRB1*11:157	0.0893
13					DRB1*11:159	0.0893
14					DRB1*12:21	0.0893
15					DRB1*14:142	0.0893
16					DRB1*15:66:02	0.0893

## 4 Discussion

NGS has been essential in the development of HLA genotyping in clinical histocompatibility due to its capacity to provide high-throughput and high-resolution genotypes, making it possible to reduce the time required to analyze a large number of samples and facilitate accurate studies. The multiplex long primer resolution can give determinant information to identify multiple existing alleles at low cost, compared to SBT, where screening different alleles requires preprograming multiplex PCR mixtures for only one locus. In addition, traditional methods such as SBT, SSP, and SSOP are often ambiguous in allele assignment, especially in heterozygotic samples, in contrast to NGS that can carry out precise allele assignment. Smith et al. described 21 novel sequences using NGS that were not detected by SSOP (30). Furthermore, long PCR amplification can cover significant targets of HLA loci; moreover, multiplex PCR permits the simultaneous amplification of multiple HLA loci in one mixture with a substantial reduction in production costs and processing times; this is particularly advantageous in clinical contexts, where meticulous and precise typing skills are paramount (31). **Table 6** presents the advantages and limitations of MP-NGS, PCT-NGS, SBT, SSP, and SSOP. Compared to other studies, this approach validated MP-NGS using two different methods: one typically used in clinical settings as SBT (which was considered the gold standard), and PTC-NGS, which is used to avoid artifacts caused by PCR amplifications at high resolution (19). Moreover, this is the first large-scale study to perform MP-NGS covering HLA-A, -B, -C, -DPB1, -DRB1, and -DQB1, which are important for transplantation proceedings. It also provides additional information on HLA allele frequencies in the Chinese population, including rare alleles. On the other hand, Chinese HLA frequency studies using NGS have focused on screening either class I (32) or class II (33), but not both.

**Table 6:** Advantages and limitations of MP-NGS, PCT-NGS, SBT, SSP, and SSOP.

Method	Advantage	Limitation
MP-NGS	• High resolution: it may identify alleles using exons and introns • Scalability: It may analyze multiple samples and genes simultaneously • New alleles detection: its high precision may describe new alleles not documented • Ambiguity reduction: it may determine complex haplotypes	• High initial cost of equipment • Experience in bioinformatic for the data analysis
PCT-NGS	• Low-quality DNA: Probe capture system can capture low-quality DNA targets • Long coverage: the system is designed to cover the most important regions of HLA loci	• Long time processing • High concentration of DNA • Pricessing costly per sample • High number of steps
SBT	• Precision: it may identify known alleles with high accuracy • It is high adopted in clinical laboratories	• Limited to specific regions (generally exons 2 and 3 in HLA class I) • Less capability identifying new alleles
SSP	• Rapid and easy: ideal for routine clinical applications • Low operating cost	• Low resolution: does not distinguish minimal differences between alleles • Limited to predefined alleles
SSOP	• High throughput: can analyze multiple samples simultaneously.	• Lower resolution: it cannot detect variations outside the designed probes • Cannot fully resolve phase ambiguities

Based on our findings, optimizing HLA genotyping by adjusting primer concentrations in multiplex pools and verifying the read depth count of this modification using NGS sequencing can improve the accuracy of genotyping organ transplantation. Therefore, determining that 100–1,000× is suitable for accurate MP-NGS genotyping, read depth values below 100× in our study showed inconsistences due to low coverage. However, the 40 samples analyzed with the optimized primer concentration yielded consistent and reliable results, thereby ensuring suitability and compatibility between donor and recipient. Statistical analysis of the Wilcoxon test determined that HLA-A and HLA-DPB1 are sensitive to primer concentration variations. However, it was relatively tedious to handle the locus HLA-A since its reads were higher in the multiplex PCR. HLA genotyping findings have determined 100–500× as an accurate range for performing this genetic analysis (34, 35), though other research has suggested depth counts of above 53× (36), considering that exceeding this threshold may generate redundant information and increase analysis time. Furthermore, it is common to have difficulties in depth control when a long amplification multiplex PCR is ongoing since it may be affected by the respective structures of these loci. It is, however, widely recognized that HLA-A is often easy to amplify because this target is more exposed compared to other loci, in contrast to loci HLA-C and DRB1, which have been reported with lower read depth (31, 37). Therefore, this outcome is encouraging since their sequencing depth was within the limit suggested, thus determining reliable results and avoiding possible complications for less donor compatibility. Moreover, this experiment showed that the time required to perform this procedure was similar to other HLA genotyping reports (38) with the advantage of analyzing a large number of samples in multiple loci at high confidence; however, there are other NGS platforms that can decrease processing time.

The accuracy of NGS results showed that our method is reliable to identify clinical histocompatibility samples, since the percentage of matches at the two- and four-digit demonstrates high reliability. It is well known that the four-digit is important for transplantation compatibility, and it is usually employed in this procedure (39); therefore, this methodology meets the minimum standard for providing sufficient information in transplant procedures. A rigorous quality control pipeline for NGS genotyped was established; however, ambiguous results were observed across all three HLA typing methodologies. For instance, the sole ambiguous HLA-A result involved an insertion detected exclusively by Sanger-based sequencing (SBT). This insertion was not identified by either multiplex PCR (MP-PCR) or probe capture-based NGS (PCT-NGS). It is plausible that PCT-NGS lacked specific probes targeting this region. However, MP-PCR yielded the same result in exon 2, suggesting that the insertion might be an artifact introduced during SBT sequencing. Furthermore, the highest variability in genotyping was observed in SBT, which typically relies on exonic regions, and it is therefore more prone to incorrect allele assignments compared to MP-NGS and PCT-NGS that amplify long amplicons. PCT-NGS results are used in parallel with MP-NGS, exposing fewer mismatches than SBT. The few ambiguities identified by MP-PCR and PCT-NGS were attributed to software-related errors. Reanalysis using an alternative bioinformatics tool, HLA-HD, produced consistent results for both MP-PCR and PCT-NGS. Furthermore, in-depth examination using IGV confirmed the presence of these sequences, indicating that the discrepancies were likely due to initial software misassignments. A total of 15 ambiguous results were identified, 5 of which involved mismatches in the two-digit. Although our MP-NGS approach employs long amplicon for its genotyping, it is possible that these discrepancies come from DNA library processing, since it is well known that enzymatic fragmentation can exhibit sequence preference, potentially cleaving near important SNPs, inducing GC-containing bias, typically observed in MHC (40); another possibility is the presence of artifacts induced by the PCR reaction, which may introduce biases into the target sequence and lead to poor assignment in allele calls (41). However, the match percentage presented for the two-digit is acceptable since it is above 98%, which is considered reliable for HLA mapping. Moreover, reports described four-digit values ≥ 95% in unambiguous calls (42). Moreover, although six-digit resolution is not commonly determined through imputation; however, the values observed using this approach were ≥95%, which indicates robustness.

This methodology may be employed in HLA populations because of its versatility in the type of allele being studied; consequently, the study with 770 patients determined an important information about the Chinese population for HLA-A, -B, -C, -DPB1, -DQB1, and -DRB1. This finding can corroborate the existing frequency information, that is, the locus HLA-A frequency of allele A*24:02:01 at 0.164, which was similarly described in the Han population by Wang et al. (43). Alleles A*11:01:01 and A*02:01:01 are commonly found in China (43, 44) in the HLA-C locus; the allele C*01:02:01 is highly identified in the Chinese population, and is reported worldwide in HLA databases. The HLA-B locus was determined as the most polymorphic locus, considering its crucial role in immune system function through the presentation of diverse peptide antigens on the cell surface of CD8^+^ T cells (45), providing genetic variability in the control of diseases. However, its variability makes transplantation search difficult for recipients and donors.

HLA class II showed a smaller number of polymorphisms where DPB1*05:01 was identified as more frequent in China (46); furthermore, studies confirm that this locus has a less polymorphic variation (47); additionally, the alleles DQB1*03:01:01 and DRB1*09:01:02 are highly reported in the Chinese population (48). Moreover, investigations on HLA-C polymorphisms across the entire gene and its flanking regions in the Chinese population have uncovered issues of significance for both clinical and evolutionary perspectives, making this allele the most polymorphic among rare alleles (49). However, further investigations are necessary to generate conclusions about HLA in the Chinese population; nevertheless, this methodology could be useful in this kind of research to provide additional information to the HLA database.

Standardization of depth of coverage is important to reduce analysis costs while still obtaining the necessary information for accurate HLA compatibility assessment. Moreover, minimizing the number of reaction mixtures per sample significantly decreases both time and cost, while the results demonstrated consistency despite the limited sample size in standardization and reliability analysis, and increasing the number of samples could yield additional insights. These six loci are the most useful when determining donor compatibility in kidney transplantation. While the initial validation was performed on a set of 70 random samples, which included both common and rare variants, the method was subsequently applied to a larger cohort of 770 individuals. Although no samples in this larger set were Sanger-sequenced due to logistical constraints, no novel alleles outside our primer coverage were observed. These results suggest that the method generalizes well to larger populations without introducing unanticipated issues. This MP-NGS opens the way for future experiments to include other loci such as DQA and DPA to provide a better comprehensive donor–recipient HLA framework; moreover, this MP-NGS approach may provide information to identify new HLA alleles in the Chinese population and support broader validation across additional ethnic groups, providing sufficient read depth with lower cost, offering a reliable method in HLA genotyping experiments.

## 5 Conclusion

This study presents an optimized NGS-based multiplexing approach for HLA genotyping. Our HLA genotyping describes depths in the range of 100–1000× and, combined with strong agreement between MP-NGS, SBT, and PCT-NGS results, proves to be a reliable method in clinical, population, and evolutionary studies. Thus, our six-site multiplex PCR method is highly valuable in simplifying and reducing methodological costs. This six-site method offers phase-unambiguous genotyping data, even with a limited sample size, and has the potential to replace conventional methods for polymorphism discovery, paving the way for future studies in various HLA populations.

## Data Availability

The datasets generated for this study are not publicly available due to legal restrictions on data sharing imposed by the Chinese government. However, the data may be made available by the corresponding author upon reasonable request.
